# Editorial: Inorganic Chemistry Editor’s Pick 2021

**DOI:** 10.3389/fchem.2021.695188

**Published:** 2021-04-28

**Authors:** Luís D. Carlos

**Affiliations:** ^1^ Phantom-g, CICECO – Aveiro Institute of Materials, Department of Physics, University of Aveiro, Aveiro, Portugal

**Keywords:** lanthanides, coordination polymers, MOFs, carbon nanotubes, oxysulfides

**Editorial on the Research Topic** **Inorganic Chemistry Editor’s Pick 2021**We are pleased to introduce the collection Inorganic Chemistry Editor’s Pick 2021.

This collection showcases 19 manuscripts (including four review papers) from the past couple of years authored by scientists of distinct countries such as Australia, Chile, China, France, Germany, India, Iran, Italy, Pakistan, Poland, Portugal, Saudi Arabia, Switzerland, and United Kingdom. The collection aims to further support Frontiers’ strong community by recognizing highly deserving authors. The selected manuscripts highlight the broad diversity of research performed across the section, including the synthesis and general characterization (e.g., structural, magnetic, optical) of coordination polymers and metal-organic frameworks (MOFs) and its application for sensing, nanoplates for supercapacitors, nanophosphors for upconversion and the dielectric properties of carbon nanotubes, for instance. The review manuscripts encompass the biomolecular modification of MOFs, bulk and nano metal oxysulfides, rhenium complexes as probes for prokaryotic and fungal cells, and atomistic simulations of rare-earth orthophosphates.

All research presented here illustrates strong advances in experiments, theory, and *in silico* with applications to compelling problems aiming to put a spotlight on the main areas of interest for the section.

